# Social credit scores reduce interpersonal cooperation and trust

**DOI:** 10.1371/journal.pone.0335810

**Published:** 2025-11-07

**Authors:** Alexander Genevsky

**Affiliations:** Rotterdam School of Management, Erasmus University, Rotterdam, Netherlands; University of Siena Library of Humanities - Arezzo: Universita degli Studi di Siena, ITALY

## Abstract

Social credit systems (SCS) are increasingly used by government agencies and private firms to assign scores to individuals based on social status and behavior. These scores subsequently impact access to social and economic opportunities, resources, and interactions. The ethical and privacy concerns of SCS are frequently overlooked due to their purported, yet unverified, social and economic benefits. In this paper, we examine the impact of social credit scores on cooperation, trust, and partner selection in economic decision-making. Contrary to the intuitive notion that social credit scores facilitate interactions by increasing transparency, we find that the availability of SCS information leads to lower trust and reduced cooperation between individuals. Additionally, we find that social credit scores create persistent biases in the perception of interaction partners, which remain resistant to change even when directly contradicted by relevant behavioral evidence. These effects disproportionately disadvantage individuals with lower scores, exacerbating existing social and economic inequalities, and demonstrate that reputational systems can amplify polarization rather than promote fairness and cooperation. These findings provide important insights for policymakers, regulators, and businesses considering the adoption of social credit systems by highlighting the potential for unintended negative consequences in both public and commercial domains.

## Introduction

Social credit score (SCS) systems assign quantifiable values to individuals based on their financial history, social behavior, and public interactions, with the stated goal of facilitating improved economic and social exchanges [1,2]. These metrics are subsequently used to offer preferential treatment to those with high scores and apply restrictions and penalties on those with low scores. While proponents of SCS argue that these systems enhance transparency and facilitate interactions, prior research suggests that reputational information can have unintended consequences, often reinforcing inequality and social exclusion rather than promoting cooperation [3,4]. Moreover, the structure and source of reputational information impacts its reliability and influence on decision-making [5,6]. These findings raise concerns about whether SCS encourages fair and productive social cooperation or instead deepens existing divisions through polarization and partner selection biases. In this paper, we investigate the effects of SCS systems on cooperation and trust in economic games in which participants interact under different conditions of SCS availability. We thus assess how SCS influences cooperation, trust, and the perception and selection of partners in economic interactions.

Social credit systems have become a reality for over a billion people around the world [7–10]. While often dismissed as a concern exclusive to far east autocracies, various instantiations of SCS have become increasingly pervasive in the western world, often taking the form of centralized repositories of personal information, review systems, and social media profiling [11]. A few notable examples of large-scale SCS systems have received popular attention (e.g., China, Chile, North Korea). Most remarkably, China has developed a SCS system since 2014 which gained widespread reach throughout the country in 2020 [12,13]. At the same time, a number of less comprehensive incarnations have become increasingly present across the globe and threaten to increase in scope and prominence [2,7,14]. It may seem unthinkable that SCS could take hold in western societies, but in fact many of their most basic principles have already arrived [11]. Insurance companies may legally profile customers using social media posts in order to determine their rates [15]. Identification scans at bars and restaurants are shared through a centralized system and used to screen patrons [11]. Governments now use protected demographic characteristics as risk factors in fraud detection [16]. Across these instances, prior social and financial behavior is used by government and commercial interests to select individuals for more (and less) favorable treatment.

In the current work, we define social credit systems as formalized frameworks that assign quantifiable scores or hierarchical tiers to individuals based on factors such as social and moral behavior, financial history, social status, and demographics. These scores are used in various contexts, including government policies, organizational regulations, and social or commercial interactions, to influence how individuals are treated. The stated objectives of SCS systems typically include providing relevant reputational information to facilitate interactions and incentivizing behaviors deemed desirable by the governing entity. The social credit systems addressed in this work are distinct from examples of reputational information and gossip studied in prior research in that they are formalized, centralized, and quantified.

In this paper, we use pre-registered experiments to assess the impact of SCS on cooperation, trust, and partner selection in economic interactions. Cooperation is integral for successful group interactions and is associated with numerous positive outcomes for individuals (e.g., stronger social bonds, better psychological health), as well as groups (e.g., greater productivity: [17–19]). However, cooperation is not universal, and often relies on interpersonal dynamics [3,20,21]. Trust, while related to cooperation, represents a conceptually distinct feature of interpersonal interactions that has been tied to wide range of economic outcomes [22–26]. Trust captures the belief that someone or something is reliable, truthful, and likely to fulfill expectations. While trust often facilitates cooperation, in some contexts cooperation can take place even in the absence of trust. We examine SCS effects across complementary paradigms that target these distinct constructs. The Public Goods Game (PGG) captures cooperative contributions to a shared resource in small groups, whereas the Trust Game (TG) separates interpersonal trust (sender’s transfer) from reciprocity/trustworthiness (receiver’s return). Using both tasks allows us to assess whether SCS depresses cooperation broadly, reduces interpersonal trust specifically, and/or alters reciprocity toward differently scored partners.

Labels assigned through social credit scoring systems can shape perceptions of individuals, leading to biased judgments and differential treatment [27]. When individuals are publicly assigned a high or low social credit score, these labels may serve as heuristic cues that simplify complex social evaluations and reinforce existing biases [28]. Neuroimaging studies further suggest that social credit labels may activate neural pathways influencing moral judgment [29]. Thus, beyond serving as a regulatory mechanism, social credit systems may shape social dynamics by perpetuating pre-existing social disparities.

Previous work on reputational systems suggest that SCS may facilitate interpersonal interactions [30–37]. On the other hand, the introduction of SCS information may introduce outgroup biases and negative perceptions that discourage transactions and limit interpersonal interactions [21,30,31,38]. For example, in systems with unequal access to information and resources, pre-existing disparities can be exacerbated, as individuals with advantages are more likely to selectively engage in interactions that further reinforce their privileged status [39]. Moreover, individuals may actively avoid interactions that pose potential social or economic risks, similar to the strategic decision-making observed in group defense contexts, where individuals weigh the costs and benefits of cooperation [40]. As a result, predictions regarding the net impact of SCS require consideration of two countervailing effects – reputational information may overcome hesitancy in previously uncertain interactions partners, but at the same time labeling partners may have a chilling effect on perceptions and willingness to interact. In effect, SCS may discourage interactions that would have successfully occurred absent the SCS.

It is well documented that individuals often rely on information about others, obtained through observed behavior, to guide future interactions [32–35,41]. However, this information is often incomplete or unavailable [42,43]. Thus, individuals must rely on external sources (e.g., gossip, reputation) as a substitute for direct experience [31,43–46]. In some contexts, the availability of third-party information has been found to increase cooperation between individuals [43,47–49]. However, reliance on reputational information can also lead to the dissolution of cooperative interactions due to overinterpretation of the available information [3].

## Study 1: Social credit and cooperation

### Study 1 methods

All studies were approved by the internal human subjects review board of the Rotterdam School of Management, Erasmus University (ETH2122−0548) and meet all applicable ethical guidelines. The consent procedure was conducted through written information, followed by participants active selection of a consent option on an electronic form. At the conclusion of the studies, participants were debriefed regarding the purpose and design of the study via written information presented on the screen.

Online participants were recruited via Amazon Mechanical Turk (n = 1,200; Appendix A in S1 File) between March 29 and April 5, 2022. Eighty-four participants were excluded for predefined exclusion criteria leaving 1,116 participants for analysis (32 failed attention check, 52 completed the survey in an unreasonably short amount of time). Sample sizes were determined by the available participant pool and financial resources.

In the first phase of the study, all participants answered a battery of questions regarding their education, professional experience, social status, moral behavior, financial history, and demographics, selected to mirror the criteria used in existing real-world SCS systems [9]. Participants were then randomized into SCS or noSCS conditions distinguished only by the availability of SCS information during the task. Participants in the SCS condition were subsequently assigned into one of three social credit score categories (High: A, Mid: B, and Low: C), ostensibly based on their survey responses. In reality, the social credit scores were randomly assigned in order to control for any inherent individual differences between participants in the SCS tiers and allow for analysis focused specifically on the availability of SCS information. In order to obfuscate the random assignment of the SCS scores, survey questions were constructed such that participant answer clustered in the middle of the scales, allowing for plausible assignment in any of three SCS tiers.

Cooperation is often studied in experimental settings using game theoretic paradigms popularized in behavioral economics, in particular the Public Goods Game (PPG [50–52]. We use the PGG to measure cooperation under shared group-level incentives. In each round of the game, participants were assigned into groups of four players and subsequently decided how much to anonymously transfer to a “public account” [53–55]. The summed contributions were then multiplied by three and redistributed equally among all members of the group, regardless of their contribution. Each player’s payout was the amount they kept for themselves plus their equal share of the public account. The amount contributed by a player is thus interpreted as a measure of cooperation. To ensure an incentive compatible experimental design, participants made transfer decisions regarding a $.50 bonus. Participants were informed that the responses from other study participants were used to determine the result on each trial and that one round would be selected at random to count for real and determine their final outcome. Participants did not receive feedback regarding the outcome of each round until the completion of the session.

Participants completed 10 one-shot rounds of the public goods game, each with different partners. In the SCS condition, during each round they were presented with their own social credit score and those of their partners. Participants completed one round with each possible combination of partner SCS, presented in a random order (i.e., AAA, AAB, AAC, BBB, BBA, BBC, CCC, CCA, CCB, ABC). To assess the impact of partners’ SCS, a numeric value was assigned to each score (A:3, B:2, C:1) and an aggregate value was calculated for each combination of partners (e.g., partner group ABC would receive a value of 3 + 2 + 1 = 6). These numeric values were only used in analyses and not presented to participants. Participants in the noSCS condition completed the task without SCS information about themselves or their partners. After completing the noSCS trials, participants in the noSCS condition subsequently completed additional trials identical to those in the SCS condition in which they received SCS information about themselves and their partners. Within-subjects analyses focused on this group, which received both SCS conditions. Between-subjects analyses contrasted participants who first received the SCS vs noSCS conditions.

### Study 1 results

Multi-level linear regression models first contrasted within-subject the average amount contributed in the SCS vs. noSCS condition and included random effects of participant. There was a significant effect of SCS information, characterized by a decrease in cooperation when SCS information was available to participants (coef. = −1.83, *t* = −4.26, df = 5509; *p* < .001). We further examined whether the impact of SCS on cooperation was a result of an increase in 0-value contributions. A contrast of the proportion of 0-value contributions across conditions was not significant (*x*^2^ = 0.46, *p* = 0.50) suggesting that while the introduction of SCS information resulted in a main effect of lower contribution amounts it did not impact the proportion of 0-value contributions. For sensitivity analysis of reported analyses see Appendix B in S1 File.

**Fig 1:**
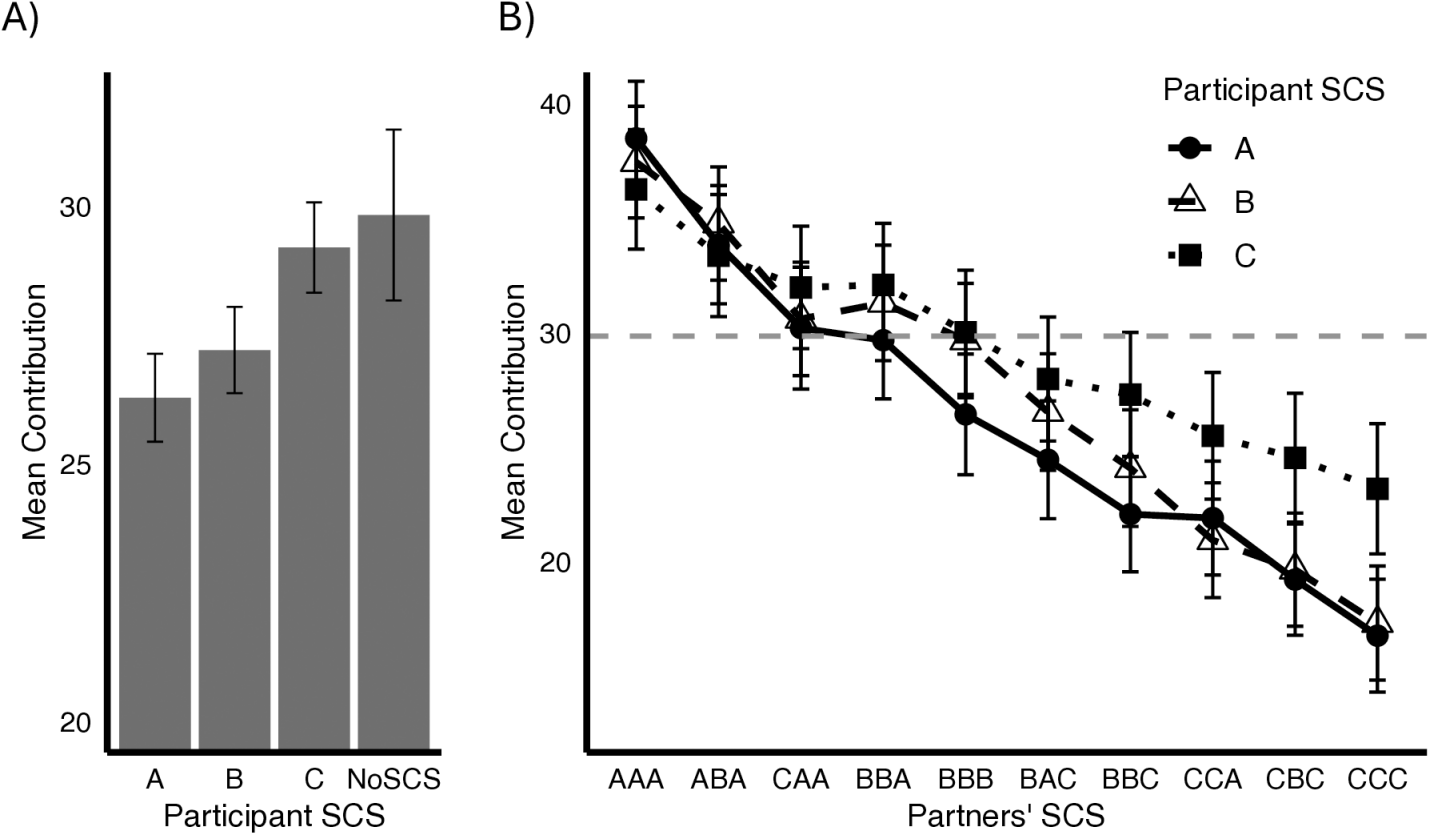
Impact of Social Credit Scores on Cooperation. Mean amounts contributed by participants in the public goods game. A) mean contributions to the public account by participant SCS; B) interaction of participant and partner SCS on contribution amount, indicating a magnified negative impact of partner SCS for individuals in higher SCS tiers. Error bars are 95% confidence intervals.

We next explored the impact of SCS on participant cooperation across the three SCS tiers. There was a significant negative linear effect of participant SCS on contributions such that higher SCS scores were associated with lower transfers to the public account (coef. est. = −2.03, *t* = −3.37, df = 1114, *p* < .001, Fig 1A). Further, participants with A and B scores contributed significantly less to the public account compared to the noSCS condition (A: *t *= −5.22, df = 4057, *p* < .001; B: *t* = −2.76, df = 4165, *p* = .006). Analyzing the impac*t* of partner SCS on participant contributions we found a strong negative linear effect of partner SCS on contributions, with reduced transfers to partners groups with lower aggregate SCS (coef. est. −2.92, *t *= −62.73, df = 10040, *p* < .001). There was also a significant in*t*eraction between participant SCS and partner SCS (*t* = 11.51, df = 10040, *p* < .001), such that *t*he negative impact of lower partner SCS on cooperation was amplified for participants with higher SCS (Fig 1B). Subsequent between-subjects analyses utilizing only the first condition presented to participants (i.e., either SCS or noSCS) replicate these findings (for details and figures see Appendix C in S1 File).

## Study 2: Social credit and trust

To separately assess interpersonal trust and reciprocity/trustworthiness in dyadic exchanges we utilize a variant of the Trust Game (TG; alternatively referred to as the Investment Game) [36,52,56,57]. On each round of the game one player (the sender) decided how much of their resources to share with their partner (the receiver). This amount was then multiplied by three, so the partner received three times the amount shared by the sender. Finally, the receiver chose how many credits (if any) to transfer back to the sender. The initial amount shared by the sender is interpreted as trust in the receiver, and any amount subsequently returned by the receiver was interpreted as an indication of their trustworthiness and reciprocity. On each round, participants made transfer decisions regarding credits that could later be exchanged to avoid a tedious matrix identification task (see Appendix D in S1 File). Participants were allocated ten credits at the beginning of the session.

### Study 2 methods

Participants (n = 781, Appendix A in S1 File) were recruited through the university student participant pool between September 25 and December 12, 2022. Eight participants were excluded for predefined exclusion criteria (see study 1) leaving 773 participants for analyses. Participants first completed the SCS questionnaire identical to study 1 and were assigned into SCS tiers as described above. They then completed seven rounds in the sender role of the trust game – 1 round with no SCS information, and then 2 rounds each with partners of A, B, and C social credit scores in a randomized order. They subsequently completed nine additional rounds in the receiver role; one round for each combination of SCS (A, B, C) and sender transfer levels (High: 10−8 credits; Mid: 7−5; Low: 4−1). Participants were informed that responses from other study participants were used to determine the result on each trial and that they were playing with different partners on each round. One round was selected at random to count for real and determined their final payment.

### Study 2 results

**Fig 2:**
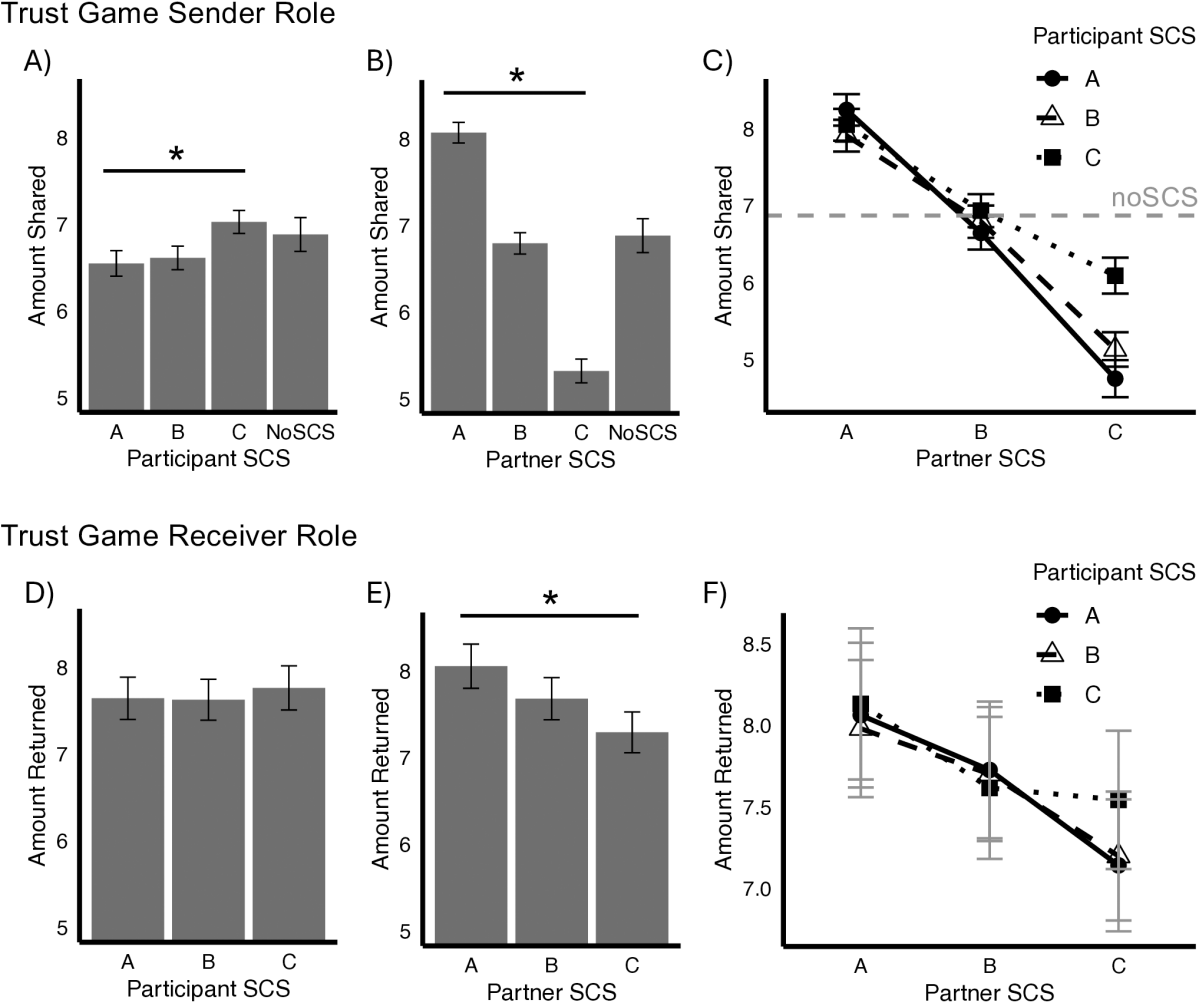
Impact of Social Credit Scores on Trust and Reciprocity. Mean amounts shared and reciprocated by participants in the sender and receiver roles of the trust game. A) mean amount shared in the sender role of the trust game by participant SCS; B) mean amount shared by senders as a function of their partner’s SCS; C) interaction of participant and partner SCS. The slope of the interaction terms indicate a magnified negative impact of partner SCS on sharing for individuals in the high SCS tier. Error bars are 95% confidence intervals. D) no significant impact of participant SCS on reciprocation. E) there is a significant linear effect of partner SCS on reciprocation of trust. Participants return more to higher SCS senders, controlling for the amount originally shared by the sender. F) reciprocation amounts broken down by participant and partner SCS. Error bars are 95% confidence intervals.

Multilevel regression models contrasted the amount shared by participants in the SCS and noSCS trials. As with cooperation, we found a significant negative effect of SCS information on amount shared, with lower average transfers in trials that included SCS information (coef. est. = −0.17, *t* = −2.31, df = 4637, *p* = 0.021). Subsequent analyses exploring the impact of the levels of participant SCS found a significant negative linear effect of SCS on the amount shared by senders, with high SCS associated with lower amounts trusted to partners (coef. est. = −0.22, *t* = −2.33, df = 816, *p* = 0.021; Fig 2A). Participants with the highest SCS level (i.e., ‘A’) shared on average significantly less with their partners (regardless of partner SCS) compared to the noSCS condition (A: *t* = 4.04, df = 2300, *p* < .001). There was also a strong linear effect of partner SCS on amount shared, with higher SCS partners receiving larger amounts (*t *= 52.79, df = 3865, *p* < .001; Fig 2B). Finally, we also found a significan*t* interaction between participant SCS and partner SCS on amount shared, further indicating that the negative impact of low partner SCS was amplified for participants with higher SCS (*t* = 11.88, df = 3862, *p* < .001; Fig 2C).

In the receiver role of the trust game, participants decided what portion of the amount they have received (the sender’s initial transfer multiplied by three) to return to the sender. We found a strong linear effect of partner SCS on reciprocated transfers, with larger amounts returned to senders with higher SCS (coef. est. = 0.38, *t* = 4.68, df = 6120, *p* < .001; Fig 2E). This finding is particularly alarming because it suggests that the amount returned to trusting senders is impacted by their SCS. In other words, individuals with lower SCS can expect less in return than those with higher SCS when making identical contributions. In addition, insofar as return transfers are an indication of the recipient’s trustworthiness, participants evidently become less trustworthy themselves when interacting with individuals with low SCS. Thus, trusting behavior is disproportionately costly for those with low scores and highlights a particularly deleterious consequence of SCS on individuals with fewer resources to share. There was no significant effect of the participant’s own SCS on return transfers (*t* = −0.51, df = 776, *p* = 0.611; Fig 2D), nor in*t*eraction between participant SCS and partner SCS (*t* = 0.82, df = 6118, *p* = 0.410; Fig 2F).

## Study 3: Partner Selection and Perception

We next assessed how SCS influences perceptions and partner selection. Participants (n = 557, Appendix A in S1 File) were recruited through the university student participant pool between September 22 and December 5, 2022. Participants first read the SCS description and public goods game instructions identical to those described above. On each trial, participants were presented with the outcome from a previously played round of the public goods game. They were able to see the contributions of three players, as well as their SCS. Participants were then asked to rate their perceptions of each player on a set of interpersonal traits, and their desire to be paired with each potential partner. Participants used seven-point scales to rate each player’s desirability, trustworthiness, strategic thinking, authenticity, generosity, competence, warmth, if they are a good person and if they are a team player. Participants were informed that their responses would be used to determine preferred partners with whom they would be paired in future rounds. There was a total of eleven rounds with participants providing ratings for a total of thirty-three partners. Across these trials participants provided ratings for a partner with every combination of SCS (A, B, or C) and contribution amount on the previous round (range $0:$10).

### Study 3 results

**Fig 3:**
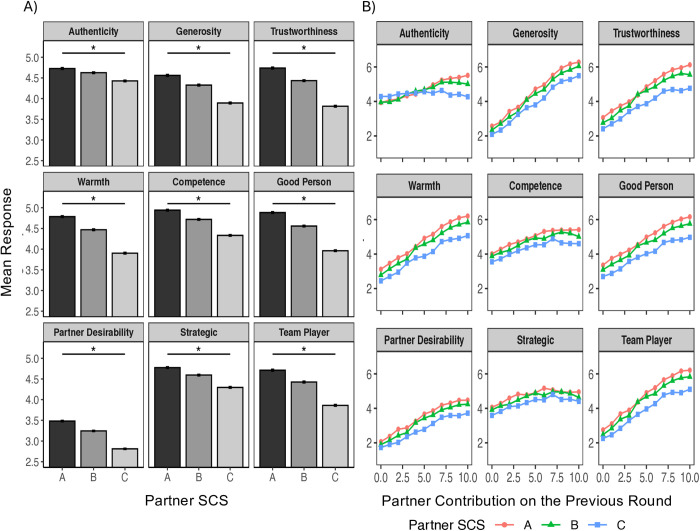
Impact of Social Credit Scores on Partner Choice and Perceptions. A) across all measured dimensions, there is a significant negative linear effect of partner SCS on participant perceptions. Analyses control for previous partner behavior, indicating these biases are resistant to the availability of objective conflicting information. B) for all traits (except for strategic thinking) there is also a significant interaction of partner SCS and previous cooperation, indicating that objective information about previous positive behavior is disproportionately discounted for individuals with lower SCS.

We first assessed the extent to which objective information about previous cooperation impacted player perceptions differently across levels of SCS. For all traits (except for strategic thinking) there was a significant interaction between previous contribution and SCS, indicating that the impact of previous contribution on perceptions was moderated by the SCS of the player (Fig 3B, Table 1). Thus, while ratings of players with low prior contributions were relatively similar across SCS tiers, larger rating differences emerged at increasing values of previous contribution. Specifically, the perceptions of partners with lower SCS received disproportionately smaller improvements in response to generous behavior than high SCS partners, despite their equivalent behavior. This finding indicates a further troubling consequence of SCS systems. Lower scores establish strong negative perceptions of individuals that are resistant to change even in the face of relevant and objectively contradictory evidence.

**Table 1:** The impacts of previous cooperation, social credit scores, and their interaction on perceptions of partners.

	[1] Partner Desirability	[2] Trustworthiness	[3] Authenticity	[4] Competence	[5] Warmth	[6] Good Person	[7] Strategic	[8] Generous	[9] Team Player
Previous contribution	0.206*** [0.006]	0.218*** [0.008]	0.066*** [0.009]	0.099*** [0.009]	0.258*** [0.008]	0.222*** [0.008]	0.091*** [0.009]	0.345*** [0.008]	0.284*** [0.008]
Social Credit Score (SCS)	0.244*** [0.016]	0.275*** [0.023]	0.272*** [0.026]	0.230*** [0.024]	0.314*** [0.022]	0.338*** [0.021]	0.255*** [0.026]	0.236*** [0.022]	0.275*** [0.023]
Previous contribution: SCS	0.019*** [0.003]	0.038*** [0.004]	0.084*** [0.004]	0.015*** [0.004]	0.026*** [0.004]	0.025*** [0.004]	−0.003 [0.004]	0.020*** [0.004]	0.030*** [0.004]
Constant	1.480*** [0.038]	2.316*** [0.056]	4.622*** [0.062]	3.563*** [0.059]	2.214*** [0.053]	2.438*** [0.052]	3.621*** [0.062]	1.864*** [0.053]	2.064*** [0.055]

Note: * p < .05, ** p < .01, *** p < .001

Regression analyses next tested the within-subject fixed effects of the partner SCS on perception ratings, controlling for the partner’s previous contributions. Differences in perceptions are independent of the objective information available regarding the player’s past cooperation. Across all measured dimensions, we find a significant effect of SCS on perceptions (Fig 3A, Table 1). For all traits, including those not directly related to social credit, players with lower SCS were associated with less favorable ratings. As expected, there was also a significant main effect of previous contribution, indicating that information about past cooperation behavior also significantly impacted perceptions of players.

## Conclusion

Social credit scores are becoming increasingly prevalent around the globe and purport to improve interactions through increased transparency and confidence. However, we find consistent evidence that the availability of SCS information decreases cooperation and trust in economic decision making. Further, we find that SCS create biased perceptions of individuals with lower scores that are resistant to change despite the availability of contradictory evidence.

Our findings align with previous work demonstrating that reputational systems can undermine cooperation when interactions are influenced by status-based preferences [4]. Specifically, the availability of SCS led to less cooperative behavior, particularly among individuals with higher SCS, who were significantly less willing to engage with lower-SCS partners. These effects mirror prior research showing that free partner choice can exacerbate inequalities by clustering individuals with similar social status together, reinforcing disparities rather than encouraging social cooperation [39]. The findings are also consistent with prior research indicating that reputational information can intensify group polarization, ultimately widening disparities between groups [3].

Additionally, our results suggest that reputational biases based on SCS information are resistant to corrective information, as lower-SCS individuals were perceived as less trustworthy even when their actual cooperative behavior contradicted these expectations. These effects bear similarity to gossip-based reputation systems, where the credibility of third-party information is influenced by self-serving biases rather than objective trustworthiness [5].

We find particularly worrying evidence that SCS has a disproportionately negative impact on individuals with lower scores, even in domains when SCS should be irrelevant. In particular, when deciding how much of a trusted amount to reciprocate, participants return significantly less to low SCS partners. This pattern of behavior suggests that individuals with lower SCS likely experience the worst of both worlds, contributing more, and receiving less in return. We also find that low SCS individuals receive systematically lower ratings across personality domains, including those unrelated to the SCS score criteria. These biases remained unchanged despite conflicting objective information regarding the actual behavior of those partners. This suggests that the biases created by SCS systems have the potential to be pervasive, far reaching, and difficult to correct.

These findings provide an illustration of the potential for unintended consequences of SCS systems. Despite concerns regarding their moral justification and legality, proponents of SCS systems often point to the benefits to society as a rationalization for their adoption. To the contrary, we show a consistent negative effect of SCS across contexts. Further, individuals with lower SCS, typically members of underprivileged communities, are disproportionately impacted. Market regulators and policy makers must carefully consider the potential harm to these communities resulting from the application of SCS systems

Our empirical studies proceed from group cooperation (PGG; Study 1) to dyadic trust and reciprocity (TG; Study 2), and then to social perception and partner selection (Study 3). This progression reflects distinct decision levels at which SCS information could impact decision making in social contexts. We thus interpret the studies as complementary evidence that SCS availability reduces cooperative contributions in groups (Study 1), is associated with lower interpersonal trust and reduced reciprocity toward low-SCS partners (Study 2), and creates persistent perception and choice biases (Study 3). Future research utilizing within-subjects designs might directly estimate mediation pathways between trust and cooperation under SCS.

It should be noted that there are elements of the empirical design utilized in these studies that deviate from some real-world examples of SCS systems. For example, participants’ SCS scores were putatively based on individual factors but were in fact randomly assigned. In addition, participants’ SCS scores remained static throughout the session and were not responsive to their behavior in the experiment. These aspects of the experiment allowed for analysis of the impact of the availability of SCS scores on interpersonal interactions, while controlling for individual differences across SCS tiers and strategic game play. Despite the empirical advantages, we also note potential limitations to generalizability. It should also be considered that our study samples are not representative of the overall population. The use of distinct sampling pools (i.e., online and university samples) that converge on similar patterns of results helps bolsters internal validity but does not directly address population-level inference. Future work should further test the external validity of SCS effects using representative samples, replications across cultural contexts, and natural field experiments where reputational labels are organically embedded in interactions.

In summary, despite the intuitive notion that social credit systems might improve interpersonal interactions, we find evidence to the contrary. We uncover several problematic issues regarding the consequences of adopting SCS systems. The introduction of SCS information decreased cooperation and trust-based behavior as well as the reciprocation of trust to lower SCS individuals. In addition, SCS information created pervasive biases, resistant to change, applying further pressure to already disadvantages group. Given the increasing prevalence of algorithmic and reputational systems, these results highlight the need for careful consideration and oversight to prevent the unintended exacerbation of social inequalities through SCS systems.

## Supporting information

S1 File Appendices. (PDF)

## References

[1] Meissner M. China’s Social Credit System: A Big-Data Enabled Approach to Market Regulation with Broad Implications for Doing Business in China. MERICS China Monit. 2017; 1–13. https://www.chinafile.com/library/reports/chinas-social-credit-system-big-data-enabled-approach-market-regulation-broad

[2] Meissner M, Wübbeke J. IT-backed authoritarianism: Information technology enhances central authority and control capacity under Xi Jinping. MERICS Pap China. 2016;1:52–6.

[3] Gross J, De Dreu CKW. The rise and fall of cooperation through reputation and group polarization. Nat Commun. 2019;10:1–10. 30770812 10.1038/s41467-019-08727-8 PMC6377668

[4] Stallen M, Snijder LL, Gross J, Hilbert LP, De Dreu CKW. Partner choice and cooperation in social dilemmas can increase resource inequality. Nat Commun. 2023;14(1):6432. doi: 10.1038/s41467-023-42128-2 37833250 PMC10575984

[5] Dores Cruz TD, van der Lee R, Bechtoldt MN, Beersma B. Nasty and noble notes: interdependence structures drive self-serving gossip. Personal Social Psychology Bulletin. 2024. 10.1177/01461672231171054 PMC11492547 37231711

[6] Testori M, Dores Cruz TD, Beersma B. Punishing or praising gossipers: How people interpret the motives driving negative gossip shapes its consequences. Soc Personal Psychol Compass. 2024;18:1–18.

[7] Shahin S, Zheng P. Big Data and the Illusion of Choice: Comparing the Evolution of India’s Aadhaar and China’s Social Credit System as Technosocial Discourses. Soc Sci Comput Rev. 2020;38:25–41.

[8] Kostka G, Antoine L. Fostering Model Citizenship: Behavioral Responses to China’s Emerging Social Credit Systems. Policy & Internet. 2019;12(3):256–89. doi: 10.1002/poi3.213

[9] Vieira dos Reis A, Press LT. Sesame Credit and the Social Compliance Gamification in China. In: Proceedings of SBGames, 2019. 270–8.

[10] Aldendorff N. Transforming society for the better? Why China’s social credit systems are surprisingly popular. 2019.

[11] Elgan M. Uh-oh: Silicon Valley is building a Chinese-style social credit system. In: Fast Company. 2019. https://www.fastcompany.com/90394048/uh-oh-silicon-valley-is-building-a-chinese-style-social-credit-system

[12] State Council. Notice of the State Council on the Planning Outline of the Construction of a Social Credit System (2014‐2020). Beijing: State Council. 2014.

[13] State Council. Planning outline for the construction of a social credit system (2014-2020) (translated). 2014. https://chinacopyrightandmedia.wordpress.com/2014/06/14/planning-outline-for-the-construction-of-a-social-credit-system-2014-2020/

[14] Mac Síthigh D, Siems M. The Chinese Social Credit System: A Model for Other Countries?. Modern Law Review. 2019;82(6):1034–71. doi: 10.1111/1468-2230.12462

[15] Baron J. Life insurers can use social media posts to determine premiums, as long as they don’t discriminate. Forbes. https://www.forbes.com/sites/jessicabaron/2019/02/04/life-insurers-can-use-social-media-posts-to-determine-premiums/?sh=ef45c9823ce1. 2019.

[16] Netherlands: End dangerous mass surveillance policing experiments. Amnesty International. https://www.amnesty.org/en/latest/news/2020/09/netherlands-end-mass-surveillance-predictive-policing/. 2020.

[17] Axelrod R, Hamilton WD. The Evolution of Cooperation. Science. 2008;211:1390–6. 10.1126/science.7466396 7466396

[18] Kollock P. Social dilemmas: The anatomy of cooperation. 1998. 183–214.

[19] Sober E, Wilson DS. Unto others: The evolution and psychology of unselfish behavior. Harvard University Press. 1999.

[20] Ahn TK, Esarey J, Scholz JT. Reputation and Cooperation in Voluntary Exchanges: Comparing Local and Central Institutions. The Journal of Politics. 2009;71(2):398–413. doi: 10.1017/s0022381609090355

[21] Tomassini M, Antonioni A. Public Goods Games on Coevolving Social Network Models. Frontiers in Physics. 2020;8.

[22] Knack S, Keefer P. Does Social Capital Have an Economic Payoff? A Cross-Country Investigation. The Quarterly Journal of Economics. 1997;112(4):1251–88. doi: 10.1162/003355300555475

[23] La Porta R, Lopez de Silanes F, Shleifer A, Vishny RW. Trust in Large Organizations. Am Econ Rev. 1997;87:333–8.

[24] Guiso L, Sapienza P, Zingales L. Trusting the Stock Market. The Journal of Finance. 2008;63(6):2557–600. doi: 10.1111/j.1540-6261.2008.01408.x

[25] Guiso L, Sapienza P, Zingales L. Cultural biases in economic exchange. Q J Econ. 2009;124:1095–131.

[26] Bloom N, Sadun R, Van Reenen J. The organisation of firms across countries. Q J Econ. 2012;127:1663–705.

[27] Fiske ST. Stereotype content: Warmth and competence endure. Curr Dir Psychol Sci. 2018;27:67–73. 29755213 10.1177/0963721417738825 PMC5945217

[28] Van Bavel JJ, Packer DJ, Cunningham WA. The Neural Substrates of In-Group Bias. Psychological Science. 2008;19:1131–9. 19076485 10.1111/j.1467-9280.2008.02214.x

[29] Delgado MR, Frank RH, Phelps EA. Perceptions of moral character modulate the neural systems of reward during the trust game. Nat Neurosci. 2005;8(11):1611–8. doi: 10.1038/nn1575 16222226

[30] Barclay P. Trustworthiness and competitive altruism can also solve the “tragedy of the commons”. Evolution and Human Behavior. 2004;25(4):209–20. doi: 10.1016/j.evolhumbehav.2004.04.002

[31] Sommerfeld RD, Krambeck H-J, Semmann D, Milinski M. Gossip as an alternative for direct observation in games of indirect reciprocity. Proc Natl Acad Sci U S A. 2007;104(44):17435–40. doi: 10.1073/pnas.0704598104 17947384 PMC2077274

[32] Wedekind C, Milinski M. Cooperation through image scoring in humans. Science. 2000;288(5467):850–2. doi: 10.1126/science.288.5467.850 10797005

[33] Jordan JJ, Hoffman M, Nowak MA, Rand DG. Uncalculating cooperation is used to signal trustworthiness. Proc Natl Acad Sci U S A. 2016;113(31):8658–63. doi: 10.1073/pnas.1601280113 27439873 PMC4978259

[34] Milinski M. Reputation, a universal currency for human social interactions. Philos Trans R Soc Lond B Biol Sci. 2016;371(1687):20150100. doi: 10.1098/rstb.2015.0100 26729939 PMC4760200

[35] Mesoudi A, Whiten A, Dunbar R. A bias for social information in human cultural transmission. Br J Psychol. 2006;97(Pt 3):405–23. doi: 10.1348/000712605X85871 16848951

[36] Brülhart M, Usunier J-C. Does the trust game measure trust?. Economics Letters. 2012;115(1):20–3. doi: 10.1016/j.econlet.2011.11.039

[37] Delgado‐Ballester E, Luis Munuera‐Alemán J. Does brand trust matter to brand equity?. Journal of Product & Brand Management. 2005;14(3):187–96. doi: 10.1108/10610420510601058

[38] Mohtashemi M, Mui L. Evolution of indirect reciprocity by social information: the role of trust and reputation in evolution of altruism. J Theor Biol. 2003;223(4):523–31. doi: 10.1016/s0022-5193(03)00143-7 12875829

[39] Snijder LL, Stallen M, Gross J. Decision-makers self-servingly navigate the equality-efficiency trade-off of free partner choice in social dilemmas among unequals. J Econ Psychol. 2024;105:102758.

[40] Snijder LL, Gross J, Stallen M, De Dreu CKW. Prosocial preferences can escalate intergroup conflicts by countering selfish motivations to leave. Nat Commun. 2024;15(1):9009. doi: 10.1038/s41467-024-53409-9 39424809 PMC11489402

[41] Romano A, Giardini F, Columbus S, de Kwaadsteniet EW, Kisfalusi D, Triki Z, et al. Reputation and socio-ecology in humans. Philos Trans R Soc Lond B Biol Sci. 2021;376(1838):20200295. doi: 10.1098/rstb.2020.0295 34601915 PMC8487743

[42] Nowak MA, Sigmund K. The dynamics of indirect reciprocity. J Theor Biol. 1998;194(4):561–74. doi: 10.1006/jtbi.1998.0775 9790830

[43] Feinberg M, Willer R, Stellar J, Keltner D. The virtues of gossip: reputational information sharing as prosocial behavior. J Pers Soc Psychol. 2012;102(5):1015–30. doi: 10.1037/a0026650 22229458

[44] Giardini F, Vilone D. Evolution of gossip-based indirect reciprocity on a bipartite network. Sci Rep. 2016;6:37931. doi: 10.1038/srep37931 27885256 PMC5122853

[45] Dores Cruz TD, Thielmann I, Columbus S, Molho C, Wu J, Righetti F, et al. Gossip and reputation in everyday life. Philos Trans R Soc Lond B Biol Sci. 2021;376(1838):20200301. doi: 10.1098/rstb.2020.0301 34601907 PMC8487731

[46] Gallo E, Yan C. The effects of reputational and social knowledge on cooperation. Proc Natl Acad Sci U S A. 2015;112(12):3647–52. doi: 10.1073/pnas.1415883112 25775544 PMC4378402

[47] Fehr D, Sutter M. Gossip and the efficiency of interactions. Games and Economic Behavior. 2019;113:448–60. doi: 10.1016/j.geb.2018.10.003

[48] Wu J, Balliet D, Van Lange PAM. Reputation management: Why and how gossip enhances generosity. Evolution and Human Behavior. 2016;37(3):193–201. doi: 10.1016/j.evolhumbehav.2015.11.001

[49] Wu J, Balliet D, Van Lange PAM. When Does Gossip Promote Generosity? Indirect Reciprocity Under the Shadow of the Future. Soc Psychol Personal Sci. 2015;6:923–30.

[50] Camerer CF. Progress in Behavioral Game Theory. Journal of Economic Perspectives. 1997;11(4):167–88. doi: 10.1257/jep.11.4.167

[51] Camerer CF, Fehr E. Behavioral Game Theory: Experiments in Strategic Interaction. Roundtable Series in Behavioral Economics. New York: Sage and Princeton University Press. 2003.

[52] Ostrom E, Walker JM. Trust and Reciprocity: Interdisciplinary Lessons from Experimental Research. New York: Sage. 2003.

[53] Ledyard JO. 2. Public Goods: A Survey of Experimental Research. The Handbook of Experimental Economics. Princeton University Press. 1995. 111–94. doi: 10.1515/9780691213255-004

[54] Chaudhuri A. Sustaining cooperation in laboratory public goods experiments: a selective survey of the literature. Exp Econ. 2011;14:47–83.

[55] Hardin G. The tragedy of the commons. Science. 1968;162:1243–8. 5699198

[56] Berg J, Dickhaut J, McCabe K. Trust, reciprocity, and social history. Games Econ Behav. 1995;10:122–42.

[57] Sapienza P, Toldra-Simats A, Zingales L. Understanding Trust. Econ J. 2013;123:1313–32.

