# Supplementary material for: Social credit scores reduce interpersonal cooperation and trust
Source: PLoS One. 2025 Nov 7;20(11):e0335810. doi: 10.1371/journal.pone.0335810 (PMC12594354; doi:10.1371/journal.pone.0335810)
Supplement: S1 File — (PDF) [file pone.0335810.s001.pdf]

Supplementary Material for:

Social credit systems decrease interpersonal cooperation and trust

Alexander Genevsky [1*]

[1] Rotterdam School of Management, Erasmus University

[*] Corresponding author

Email: genevsky@rsm.nl (AG)

Appendix A

Participant Descriptives

	Exp. 1 (n=1116)		Exp. 2 (n=781)		Exp. 3 (n=557)	
	n	%	*n*	%	*n*	%
Age (mean, sd)	41.15 (12.26)		19.17 (1.64)		18.67 (1.83)	
Gender						
Female	534	47.8%	354	45.3%	209	50.1%
Male	576	51.6%	426	54.5%	208	49.9%
Non-binary	0	0%	0	0%	0	0%
Declined	6		1		0	
Socio-economic level						
Lower	165	14.8%	25	3.2%	6	1.5%
Lower-middle	336	30.0%	50	6.4%	25	6.1%
Middle	483	43.2%	199	25.5%	116	28.5%
Upper-middle	126	11.2%	372	47.6%	209	51.4%
Upper	6	0.5%	135	17.3%	61	15.0%

Note: In study 3 demographic details were obtained from 417 of 557 participants due to technical issues.

Appendix B

Sensitivity Analysis

In order to determine the minimal effect detectable with the achieved sample size we conducted sensitivity analyses using simulated data. For each analysis, at each level of possible effect magnitude, we simulate subject and trial level data utilizing the intercept and error distributions observed in the original data. We then simulate data over one thousand iterations, only manipulating the magnitude of the treatment effect. Finally, we assess the proportion of iterations in which a significant difference between conditions was found ($p < .05$) to determine the observed power.

Figure S1a.

Sensitivity analyses of the main effect of the availability social credit scores on contributions in the Public Goods Game (Study 1).

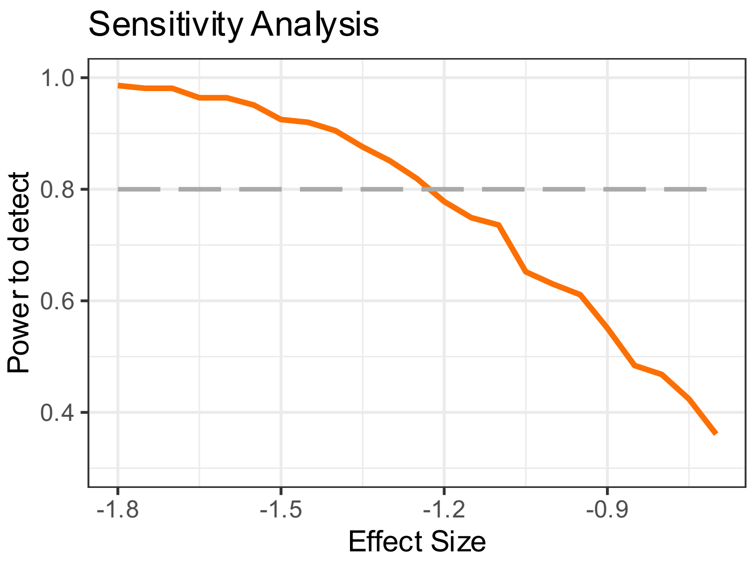

Figure S1b.

Sensitivity analyses of the linear effect of participant SCS on contributions in the Public Goods Game (Study 1).

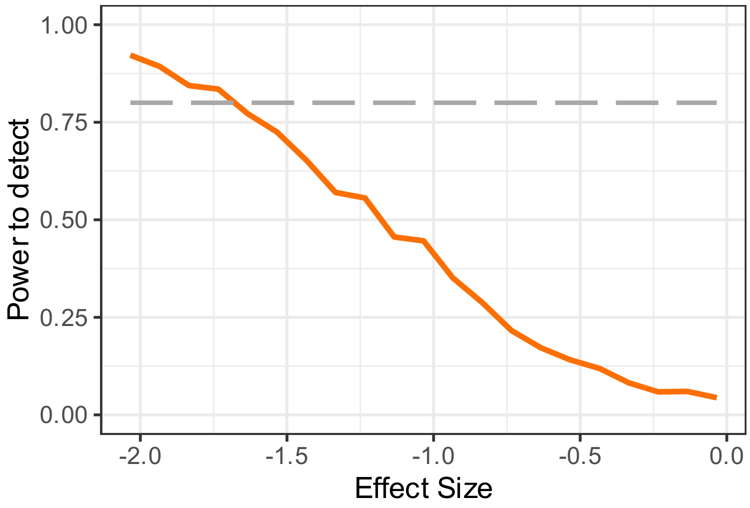

Figure S1c.

Sensitivity analyses of the main effect of the availability social credit scores on amounts trusted by participants in the sender role in the Trust Game (Study 2).

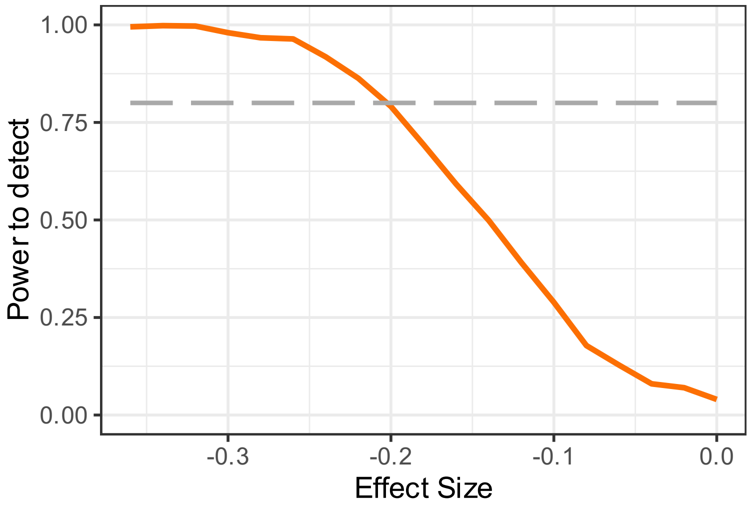

Figure S1d.

Sensitivity analyses of the linear effect of participant SCS on the amounts sent by participants in the Trust Game sender role (Study 2).

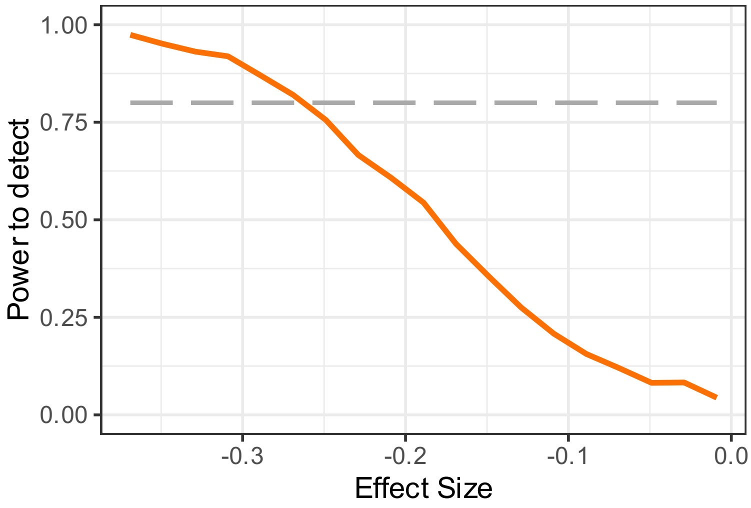

Figure S1e.

Sensitivity analyses of the linear effect of partner SCS on the amounts returned by participants in the Trust Game receiver role (Study 2).

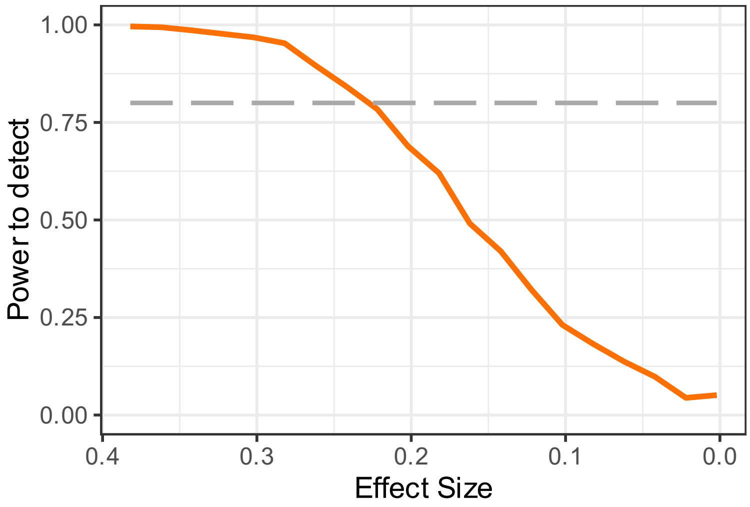

Appendix C

Study 1: Between-subjects analysis

Results

A nested linear regression model including random effects of subject contrasting trials with and without social credit score information indicates a significant decrease in transfers when social credit information is available to participants (t = -2.27, p = .034). This effect is independent of the content of the social credit score information. High social credit scores are associated with lower transfers to the public account (Fig. S2A). Participants with A social credit scores transfer significantly less to the public account compared to the no social credit information condition (A: t = -2.45, df = 559, p = .014). Participants with B social credit scores also transfer a marginally significantly smaller amount to the public account when SCS information is available (t = -1.83, df = 548, p = .067). To assess the impact of the social credit scores of their partners on participant transfers, a numeric value is assigned to each social credit score (A:3, B:2, C:1) and an aggregate value is calculated for each combination of partners (e.g., partner group ABC would receive a value or 3+2+1=6). There is a strong negative linear effect of aggregate partner credit score value on transfer size (t = -48.78, df = 5084, p < .001), with lower transfers to groups with lower aggregate social credit scores (Fig. 2A). Finally, when considered together, there is a significant interaction between participant social credit scores and their partners' scores (t = 8.84, df = 5083, p < .001). Specifically, the negative impact of declining partner scores on transfers is amplified in participants with higher social credit scores (Fig. S2B).

Figure S2

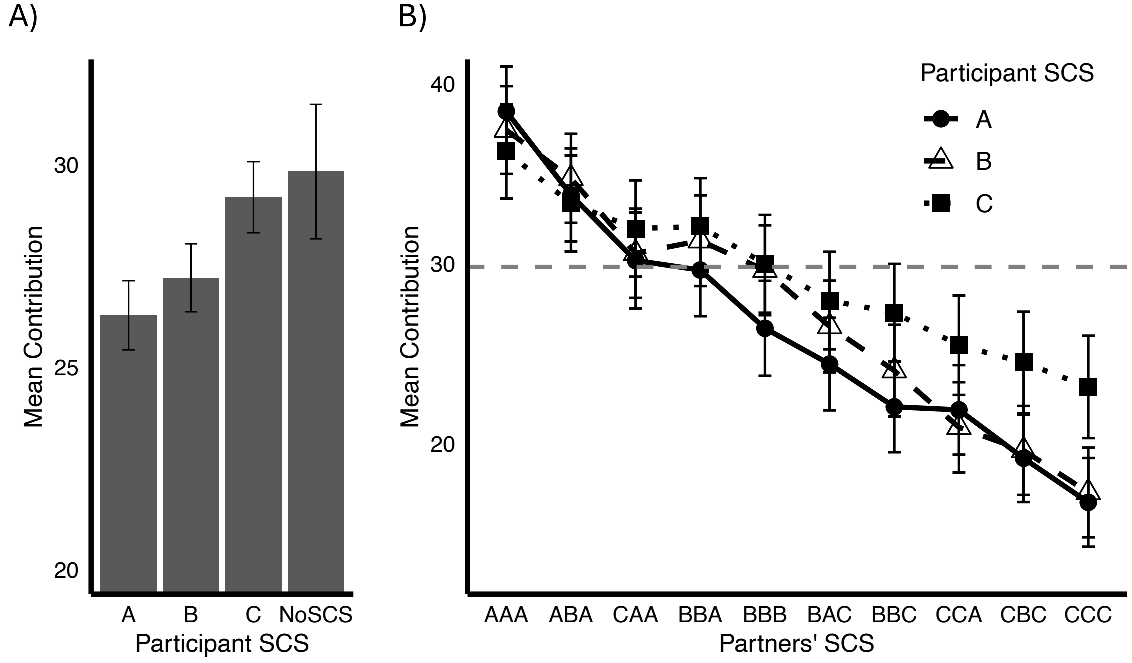

Study 1: Impact of Social Credit Scores on Cooperation (Between-Subjects Analysis)

Participants contributions to a shared public account in the public goods game. A) mean contributions to the public account by participant SCS; B) interaction of participant and partner SCS indicating a magnified negative impact of partner SCS for individuals in higher SCS tiers. Error bars are 95% confidence intervals.

Appendix D

Sample matrix identification task

Participants must accurately indicate the number of 0’s in the matrix to continue.

0	1	0	1	0	1	0	1	1	0	0	1	0	0	0
0	1	0	1	0	1	0	1	0	1	1	0	0	1	0
1	0	1	1	1	0	0	0	1	1	0	0	0	1	0
1	1	0	1	0	1	0	0	1	0	0	1	0	1	1
0	0	1	1	0	1	0	1	0	0	1	0	1	1	0
0	1	0	1	0	1	0	0	0	1	0	1	0	1	1
1	1	1	0	1	1	1	1	1	1	1	0	1	0	0
0	0	1	1	0	0	0	1	0	1	0	1	1	0	1
0	1	1	1	1	0	0	1	0	1	0	1	0	0	0
1	0	0	0	1	0	1	0	1	0	0	1	0	1	1

Appendix E

Study materials

Survey questions putatively used to assign participant social credit scores

First, please answer a few questions about yourself.

Please be as thoughtful and honest as you can.

How does the size of your friend group compare to others you know?

- ○ One of the largest
- ○ Above average
- ○ Average
- ○ Below average
- ○ One of the smallest

In the past year how often have you volunteered to help other people?

- ◯ A great deal
- ◯ A lot
- ◯ A moderate amount
- ◯ A little
- ◯ Not at all

To what extend have you been dishonest over the last year?

- ◯ A great deal
- ◯ A lot
- ◯ A moderate amount
- ◯ A little
- ◯ Not at all

How much debt would you say you have had in your life? (credit card debt, student loans, care loans, etc.)

- ○ A great deal
- ○ A lot
- ○ A moderate amount
- ○ A little
- ○ None at all

Have you even missed a debt payment? (for example a credit card payment)

- ○ Yes, a number of times
- ○ Yes, a few times
- ○ Yes, once
- ○ I don't think so
- ○ Definitely not

Have you ever stolen anything?

- Yes I have
- Yes, but only small things
- I don't think so
- Definitely not
- Not sure

What percentage of your income would you say you give to charity annually?

- 0%
- 1% to 2%
- 3% to 5%
- 6% to 10%
- 11% to 15%
- 16% to 20%
- 21% to 25%
- more than 25%

How would people that know you respond to these questions about you?

	Not at all	A little	Moderately	Very	Not sure
How honest are you?	○	○	○	○	○
How reliable are you?	○	○	○	○	○
How likely are you to lie to cover up a mistake?	○	○	○	○	○
How friendly are you?	○	○	○	○	○
How ambitious are you?	○	○	○	○	○
How likely are you to have debt?	○	○	○	○	○

Task instructions and presentation.

SCS score presentation

In these rounds, you and your partner will both have a little more information about each other.

Page Break

Social Credit Score A Social Credit Score System is a way to measure an individual's reputation in their community. The Social Credit Score is established to track and evaluate the trustworthiness of individuals.

The scores range from **A (the highest)** to **C (the lowest)**

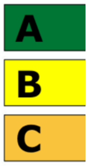

Page Break

Social Credit Score
The questions you answered earlier are used to determine the Social Credit Scores.

Based on your responses you have been assigned a Social Credit Score of **{A/B/C}**

Your Social Credit Score

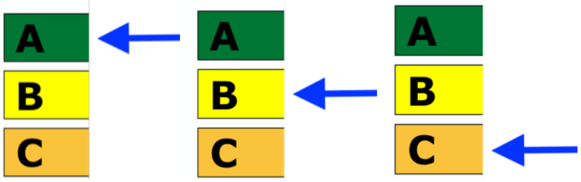

Cooperation task instructions and presentation

Instructions

You have received **10 credits**.
Each of the other 3 participants in your group has **also received 10 credits**. **Your task is to decide how many credits to transfer to the 'public account'**. You can transfer any amount between 0 and 10. Each of the other 3 participants will also make a decision about their transfer to the public account.

After all 4 participants in your group have reached a decision, the transfers to the public account are summed. Then, **this sum is multiplied by 3**. Finally, the multiplied sum in the public account is **divided equally between the 4 participants in your group**.

Your final payoff will be: The amount you did not contribute + your equal share of the public account

---

Now you will complete **10 rounds**.

One of these rounds may also be selected at random to determine your number of credits.

In these rounds, you will have more information about the other players.

Your Score: **{A/B/C}**

The other members of your group have received the following scores:

Member 1: **{A/B/C}**

Member 2: **{A/B/C}**

Member 3: **{A/B/C}**

Please indicate how much you want to transfer to the public account.

	0 1 2 3 4 5 6 7 8 9 10
Your transfer to the public account	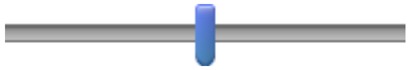

{In the noSCS condition SCS information is not presented}

Trust task instructions and presentation

Instructions

1. You will be randomly and anonymously paired with another participant

2. Within each pair, one participant is assigned to be the Sender, while the other participant is assigned to be the Returner.

3. To start, the Sender receives 10 credits and the Returner receives 0 credits. The Sender can send some or all of their 10 credits to the Returner.

4. The amount the Sender transfers to the Returner is multiplied by 3, so the Returner receives 3 times as many credits.

5. Once the Returner receives the tripled amount they can then decide to send any amount of it back to the Sender.

6. The Sender's final number of credits will be the amount they kept + the amount the Returner sends back. The Returner's final payoff is the amount the Sender's transfer * 3 - the amount they transfer back to the Sender

<u>Let's look at an example:</u>
If the Sender transfers **7** credits to the Returner, that amount will be tripled and the Returner will receive **21** credits.
The Returner can now decide to transfer back to the Sender any amount from **0 to 21** credits.
The Sender's total credits will be the 3 credits they decided to keep **plus** whatever the Returner sends back.

Page Break

Great. Now you will complete **6** rounds. **One of these rounds will be selected at random to determine your number of credits**.

Page Break

On each of the **6 rounds**, you will be **matched with a different Returner.**

You will be able to see the Social Credit Score of the Returner you have been paired with on each round.

One of these rounds will be selected at random to determine your number of credits. You will see the result of the selected round at the end.

Your Social Credit Score: **{A/B/C}**

Returner's Social Credit Score: {A/B/C}

You have 10 credits. The amount you transfer will be multiplied by 3, so the Returner will receive 3 times the amount you transfer.
Then, the Returner can send back to you any portion of what they have received.

How many credits will you send to the Returner?

Please enter an amount from 0 to 10:

{Question repeated 6 times)

Start of Block: Trust Returner Instructions

Ok great.

In the next set of questions, you have been assigned to the **Returner role**.
On each of the next **9 rounds**, you will be **matched with a different Sender.**

On each round, you will see how many credits the Sender has sent you. That amount is then multiplied by 3.

You must then decide how many credits you would like to send back to the Sender

You will be able to see the Social Credit Score of the Sender you have been paired with on each round.

Click below to continue...

Your Social Credit Score: **{A/B/C}**

Sender's Social Credit Score: **{A/B/C}**

This Sender has sent you {x = 1-10} credits. This amount has been multiplied by 3, so **you now have {x * 3} credits.**

How many credits will you send back to the Sender?

Please enter an amount from 0 to {x * 3}:

____

Demographics collection

Start of Block: demographics

Please tell us a little more about yourself:

Age:

____

Gender

- ○ Male
- ○ Female
- ○ Non-binary
- ○ Prefer not to answer

Please select the option that most closely describes your household income while growing up

- ○ Lower Income
- ○ Lower Middle Income
- ○ Middle Income
- ○ Upper Middle Income
- ○ Upper Income
